# The Interactions Between Three Typical PPCPs and LDH

**DOI:** 10.3389/fchem.2018.00016

**Published:** 2018-03-05

**Authors:** Erwei Li, Libing Liao, Guocheng Lv, Zhaohui Li, Chengxue Yang, Yanan Lu

**Affiliations:** ^1^Beijing Key Laboratory of Materials Utilization of Nonmetallic Minerals and Solid Wastes, National Laboratory of Mineral Materials, School of Materials Science and Technology, China University of Geosciences, Beijing, China; ^2^Geosciences Department, University of Wisconsin–Parkside, Kenosha, WI, United States

**Keywords:** Cl-LDH, PPCPs, adsorption, electrostatic interaction, anion exchange

## Abstract

With a layered structure, layered double hydroxide (LDH) has potential applications in remediation of anionic contaminants, which has been a hot topic for recent years. In this study, a Cl type Mg-Al hydrotalcite (Cl-LDH) was prepared by a co-precipitation method. The adsorption process of three pharmaceuticals and personal care products (PPCPs) [tetracycline (TC), diclofenac sodium (DF), chloramphenicol (CAP)] by Cl-LDH was investigated by X-ray diffraction (XRD), Zeta potential, dynamic light scattering (DLS), BET, Fourier transform infrared (FTIR) spectroscopy, and molecular dynamics simulation. The results showed that the adsorption equilibrium of TC and DF could be reached in 120 min, and the maximum adsorption capacity of the TC and DF were 1.85 and 0.95 mmol/g, respectively. The isothermal adsorption model of TC was fitted with the Freundlich adsorption model, and the isothermal adsorption model of DF was fitted with the Langmuir adsorption model. The adsorption dynamics of TC and DF followed the pseudo-second-order model. The adsorption mechanisms of the three PPCPs into Cl-LDH were different based on the experimental results and molecular dynamics simulation. The TC adsorption on Cl-LDH was accompanied by the electrostatic interactions between the negative charge of TC and the positive charge of Cl-LDH. The uptake of DF was attributed to anion exchange and electrostatic interaction. Cl-LDH does not adsorb CAP due to no electrostatic interaction. The molecular dynamic simulation further confirmed different configurations of three selected PPCPs, which were ultimately responsible for the uptake of PPCPs on Cl-LDH.

## Introduction

For the past decades, a wide range of pharmaceutical and personal care products (PPCPs) have been repeatedly observed in natural waters all over the world (Richardson et al., 2005; Nakada et al., 2007; Sui et al., 2015). Owing to their bioaccumulation and biological activity, possible impacts of PPCPs on the human health and ecological safety have received a lot of concerns (Suárez et al., 2008; Sun et al., 2016). Due to the high demands of PPCPs in curing diseases and sustaining economic development in agriculture and livestock farming, the global production of PPCPs was over 1 × 10^6^ tons in 1993, and the production keep increasing. Among PPCPs, Tetracycline (TC) was reported as the most widely used in animal feed. However, the majority of used TC was hard to be metabolized by animals or human beings, resulting in a considerable amounts as high as 80–90% of initial value or its metabolites being released to the external environment (Ahmadi et al., 2017). The environmental concentrations of TC was about 1.12 μg/L (Jie and Shuhe, 2015). Another widely used non-steroidal anti-inflammatory drug was Diclofenac sodium (DF), which had potential threats in municipal wastewaters and surface waters (Huang et al., 2017). The environmental concentrations of DF could reach 28.4 μg/L (Qi et al., 2015). As a broad-spectrum antibiotic, chloramphenicol (CAP) was widely used in both animal and human medicine. Nevertheless, CAP had severe side-effects on humans, such as bone marrow suppression, aplastic anemia acute leukemia, “gray baby syndrome” etc. (Sai et al., in press). Therefore, environmental pollution caused by abuse of PPCPs is becoming more and more serious. In order to eliminate the potential risk of the PPCPs, it is necessary to get rid of the PPCPs in natural waters. Accordingly, many methods have been developed to remove the PPCPs, such as physical (Kim and Tanaka, 2009), chemical (Huber et al., 2005), and biological treatments (Suarez et al., 2010).

Adsorption is an old, simple yet effective method. Eco-friendly adsorbents that adsorption process could remove hazardous chemicals efficiently without special facilities and unknown byproducts has been successfully utilized for the removal of antibiotics from aqueous solutions. Adsorbents for antibiotics removal, such as silicate (Sun et al., 2017), clay minerals (Lv et al., 2017), mesoporous materials (Zhang et al., 2015; Li et al., 2017), and layered double hydroxides (LDHs) (Monash and Pugazhenthi, 2014) have been extensively studied. LDHs constitute an important class of layered materials, which have potential applications in remediation of anionic contaminants (Prasanna and Vishnu Kamath, 2008). The chemical formula can be typically expressed as “[M(II)_1−*x*_M(III)_*x*_(OH)_2_]^*x*+^(A^*n*−^)_*x*/*n*_•mH_2_O,” in which the M(II) and M(III) are divalent and trivalent cations, represented by Mg^2+^ and Al^3+^ or many other transition metal cations (Jobbágy and Regazzoni, 2011; Peng et al., 2015). A^*n*−^ stands for an interlayer anion with a negative charge n, and m represents the number of interlayer water molecules. The value of *x* has a range of 0.17 and 0.33. Many anions or anionic complexes (both organic and inorganic) can be incorporated into the crystal structure of LDHs. The LDHs can be abbreviated as [M^II^-M^III^-A], where M^II^ can be occupied by Zn^2+^, Mg^2+^, Cu^2+^, Mn^2+^, and Ca^2+^ ions; M^III^ stands for Al^3+^, Cr^3+^, Fe^3+^, and Ga^3+^; A^*n*−^ refers to Cl^−^, ${\text{NO}}_{3}^{-}$, ${\text{CO}}_{3}^{2-}$, and ${\text{SO}}_{4}^{2-}$ (Liao et al., 2012). Due to their high anionic exchange capacities, especially, the anions are mainly extracted through ionic exchange with anions in the interlayer of LDHs. On the other hand, adsorption performance of the LDHs is affected by the surface properties of the LDHs (surface area, micropore surface area, and pore size distribution). LDHs have been considered as one of the most promising adsorbents for removing antibiotics. Thus, Cl-LDH could be a useful adsorbent to remove PPCPs.

In this study, we synthesized Cl-LDH by co-precipitated method and applied them as adsorbents for the removal of the three PPCPs (TC, DF, and CAP) from aqueous solution; to investigate the effect of pH, adsorbents time and PPCPs concentration; to discuss the adsorbent mechanism of PPCPs on Cl-LDH by theoretical calculations. In addition, the molecular dynamics simulation was also carried out to reveal the configuration of PPCPs in the interlayer space and the interactions between PPCPs and the layer structure of Cl-LDH, which helped to understand the adsorption mechanism of Cl-LDH. The purpose of this paper was to explore the possibility of using Cl-LDH for the removal of TC and DF and develop eco-friendly adsorbents.

## Methods and experiment

### Preparation of Mg-Al-Cl LDH

All reagents used in this study were of analytical grade and used without further purification. The Cl-LDH was synthesized through a co-precipitated method based on a previous study (Vreysen and Maes, 2008; Yue et al., 2017). Stoichiometric ratio of MgCl_2_•6H_2_O (24.396 g, 0.12 mol) (Beijing Chemical Works, AR) and AlCl_3_•9H_2_O (7.2429 g, 0.03 mol) (Beijing Chemical Works, AR) were dissolved in 150 mL deionized water, marked as solution A. NaOH (12.0 g, 0.3 mol) (Beijing Chemical Works, AR) and NaCl (1.7532 g, 0.03 mol) (Beijing Chemical Works, AR) were dissolved in 150 mL boiled deionized water to form solution B. Solution A and solution B were added dropwise using a burette at a rate of two drops per second into a three-necked flask simultaneously, and the mixture was stirred to be homogeneous and the pH-value was kept around 10. The slurry was aged for 12 h at 70°C. After being centrifuged, the precipitate was collected and washed with deionized water for several times, and then LDHs sample was obtained after drying at 110°C for 24 h. During the entire process of the experiment, an inert atmosphere of N_2_ was used to prevent the influence of carbonate ions.

### Adsorption experiments

0.015 g Cl-LDH and 25 mL PPCPs (pH = 7) solution were mixed in a 50 mL centrifuge tube for all batch studies. For the isotherm study, the initial concentrations of TC (Shanghai Yiji Industrial Co., AR), DF (Chizhou Kelon Import and Export Co., AR), and CAP (Shenzhen Shijin Valley Technology Co., AR) solutions varied from 0.03 to 3.0 mmol/L. The kinetic study of PPCPs (pH = 7) was conducted at initial concentrations of 2.4 mmol/L for all the three PPCPs. For the pH study, same initial concentrations of PPCPs solutions with different pH-values (3, 4, 5, 7, 9, 11) were prepared to investigate the effect of pH on the adsorption characteristics of PPCPs. The mixtures were shaken in a water bath (CHA-S) with a speed of 150 rpm at room temperature for 24 h except for the kinetic study in which mixtures were shaken from 1 to 1,440 min. After that, the mixtures were centrifuged by high speed centrifuge (TG18K-1, DongWang instrument) at 4,500 rpm for 5 min. Then, the supernatant solutions were analyzed using the UV/VIS spectrophotometer (T6 New Century) to determine the equilibrium PPCPs concentration. PPCPs removal was identified by the difference between the initial and final solution concentrations. All batch experiment was run in duplicates.

### Methods of analyses

The structures of the samples were analyzed using a Rigaku D/Max-IIIa X-ray diffraction analyses diffractometer (XRD) with Ni-filtered CuKα radiation at 30 kV and 20 mA. Samples were scanned from 3 to 70° (2θ) at 8°/min with a scanning step of 0.01°/step. A 1° divergent slit and scatter slit and 0.3 mm receiving slit were used.

Fourier transform infrared (FTIR) spectra were collected on a Nicolet-560 spectrometer (Thermal Nicolet Co., USA) from 400 to 4,000 cm^−1^ with a nominal resolution of 4 cm^−1^. For each spectrum 16 runs were collected and averaged. The hydrotalcite samples for FTIR measurement were prepared by adding approximately 1 wt% of the sample powder to dry KBr powder, and the mixture was pressed into tablet.

The equilibrium PPCPs concentrations were analyzed by a UV/VIS spectrophotometer (T6 New Century). The optimum wavelength of TC, DF, and CAP were 357, 276, and 278 nm, respectively.

Analyzer dynamic light scattering (DLS) and Zeta-potential of the material were studied on zeta-potential (ELSZ-1000, Otsuka Electronics Co., Ltd., Japan). The BET specific surface area was determined by surface area analyzer (ASAP 2020HD, Micromeritics Instrument Corporation, USA).

## Results and discussion

### Characterization of CL-LDH

**Figure 1:**
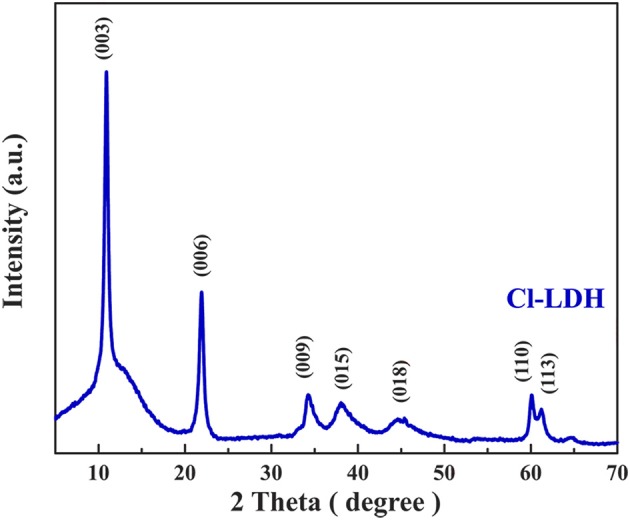
X-ray diffraction pattern of Cl-LDH.

Figure 1 showed the XRD pattern of the as-prepared Cl-LDH sample. It was apparently that a series of symmetric (00*l*) peaks at lower 2θ-values of Cl-LDH could be well-indexed to clay minerals containing layered structure LDHs. The main strong peak neared 2θ = 11.3°, corresponding to *d* = 0.7852 nm, and the other sharp peak neared 2θ = 22.7°, corresponding to *d* = 0.3920 nm, were attributed to basal reflections. The sharpness and intensity of the peaks indicated a highly crystalline structure of Cl-LDH. The typical *d* (003)-value of Cl-LDH was 0.7852 nm, which agreed well with the values reported by other researchers in the literature (Özgümüş et al., 2013; Peng et al., 2015).

The zeta potential of Cl-LDH was 36.2 mV, indicating that Cl-LDH had a good dispersity in water (Liu et al., 2017; Li et al., 2018). The DLS method resembled the size distribution of Cl-LDH in the range of 200–620 nm. The SEM image (Figure S1) of as prepared Cl-LDH reproved the size distribution of LDH as well. The BET surface of Cl-LDH was 34.85 m^2^/g, indicating that Cl-LDH had a strong adsorption ability.

### Effect of pH

**Figure 2:**
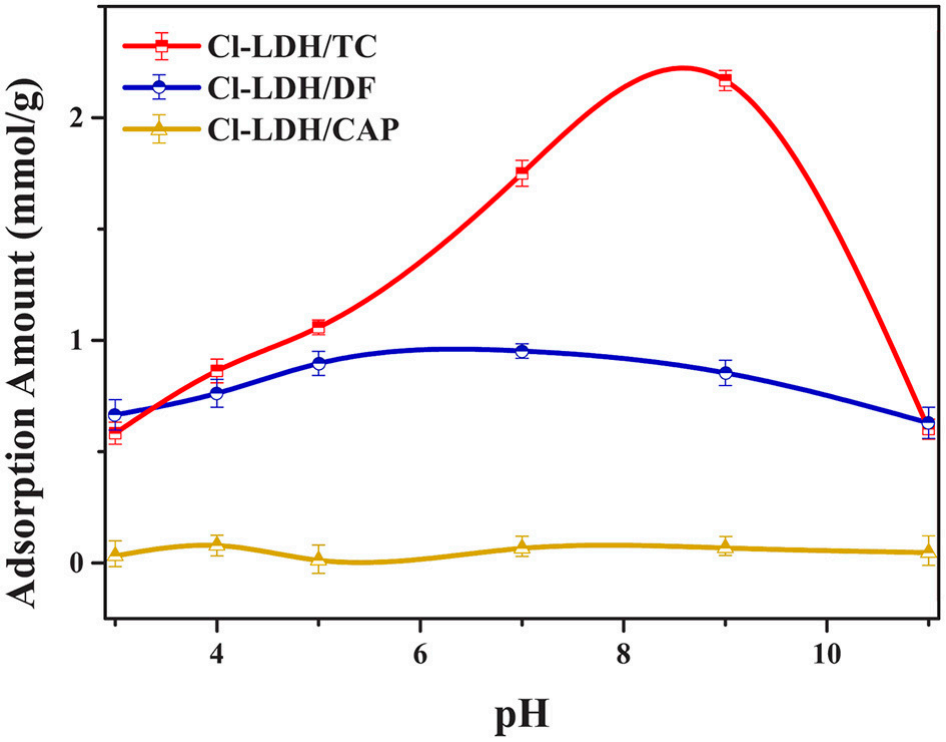
Effect of pH on adsorption amount.

**Figure 3:**
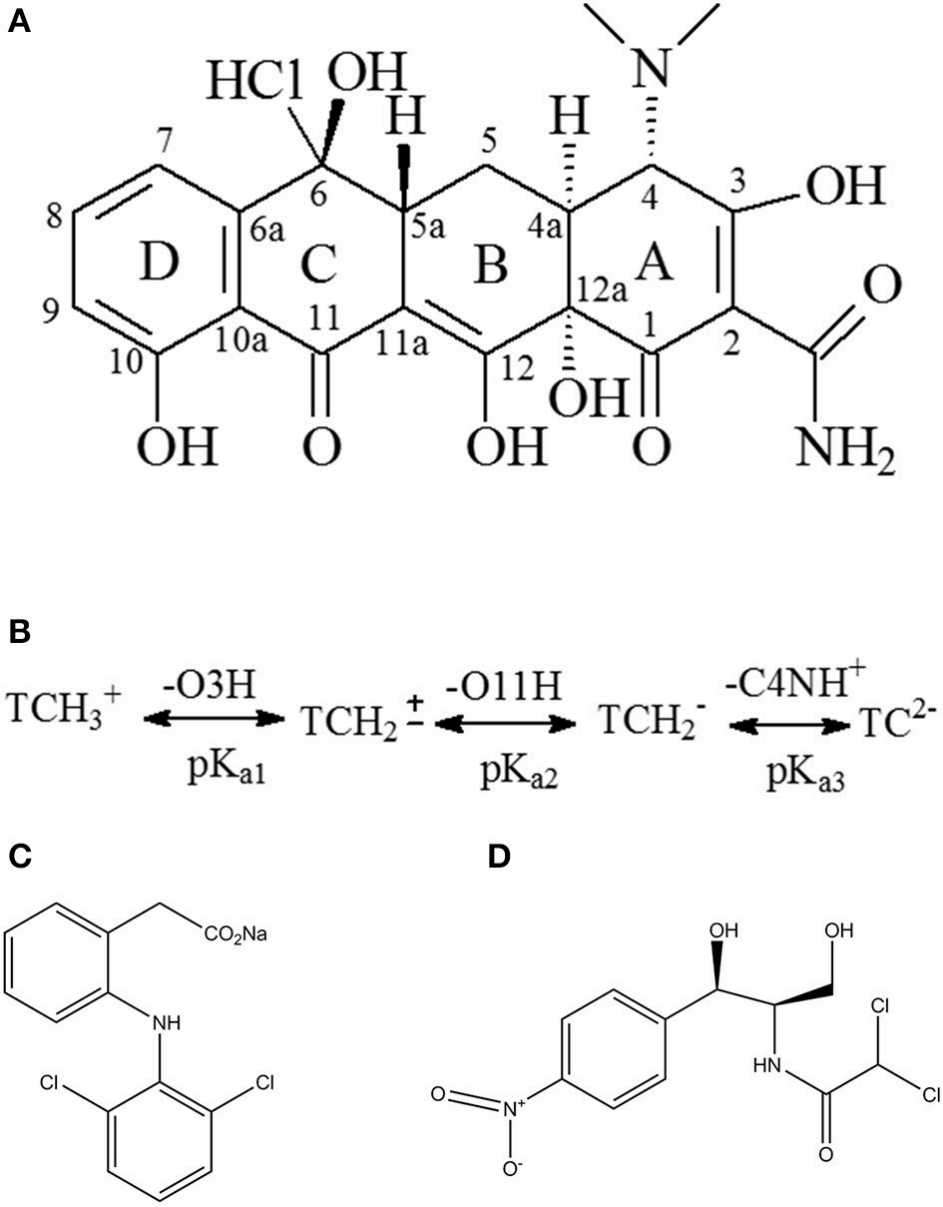
Chemical structure of tetracycline **(A)**, diclofenac sodium **(C)**, chloramphenicol **(D)**, and scheme of TC protonation/deprotonation **(B)**.

The pH of the solution should be considered to be an important factor affecting the adsorbate process due to its impact on the degree of ionization of pollutant species and the surface charge of the adsorbent (Figure 2). When pH < pH_*PZC*_, the surface of Cl-LDH (pH < 9.6) was positively charged due to isomorphic substitution of Mg^2+^ ions by Al^3+^ ions. The zeta potential value of Cl-LDH was 36.2 mV which was measured at pH around 7, suggesting positively charged surfaces. TC was a yellow crystalline substance with a molecular weight of 480.9 g/mol, and the structure of TC was given in Figure 3A. In aqueous solutions three different groups of the molecule could undergo protonation–deprotonation reactions according to varying pH of solution. As shown in Figure 3B, three values of the dissociation constants (pK_a1_ ≈ 3.4; pK_a2_ ≈ 7.5; pK_a3_ ≈ 9.3) had been determined experimentally that corresponds to existence of the protonated (${\text{TCH}}_{3}^{+}$), neutral zwitterionic (${\text{TCH}}_{2}^{\pm }$), monoanionic (TCH^−^), and dianionic (TC^−2^) forms of TC (Sassman and Lee, 2005; Soori et al., 2016). It could be easily seen that the bigger the pH-value was, the more amount of anionic species was. When the pH-value of equilibrium solution was < 9, the adsorption amount of TC increased quickly with increasing pH. The maximum adsorption amount of TC was 2.16 mmol/L when pH = 9, which was attributed to the electrostatic interactions between the negative charge of TC and the positive charge of Cl-LDH. Whereas when pH > pH_*pzc*_, namely, pH > 9.6 for Cl-LDH, the surface charge of Cl-LDH became negative and repulsive to the negatively charged TC species, which caused the result that the adsorption amount decreased with the increasing of pH.

The adsorption capacity at pH-values lower than the pKa (4.2) of DF was smaller than that at pH of 5.0, because DF was in the form of neutral molecules (–COOH) (Liu et al., 2017; Sun et al., 2017) and the structure of DF was given in Figure 3C. On increasing the pH from 5.0 to 9.0, the adsorption capacity reached maximum and kept constant due to DF in the form of anions (–COO^−^) at pH > pKa, finally decreased at pH > 9.0. The zeta potential of the Cl-LDH indicated that there was a positive surface charge on Cl-LDH at pH < 9.6, but surface positive charge decreased at pH > 9.6. This increases the electrostatic attraction between the negatively charged DF and Cl-LDH, which results in increased adsorption at pH < 9.6. Correspondingly the electrostatic attraction decreases due to the decrease of surface positive charge of Cl-LDH from 9.6 to 11 resulting in the decreased adsorption. The results suggested that the electrostatic interactions between anionic DF and cationic Cl-LDH could be the main driving forces for adsorption.

For CAP that the structure of CAP was given in Figure 3D, no uptake was observed on Cl-LDH. In the water solution, CAP molecule existed as a cation and was positively, so Cl-LDH had no electrostatic interaction with CAP.

### Cl-LDH sorption isotherm

**Figure 4:**
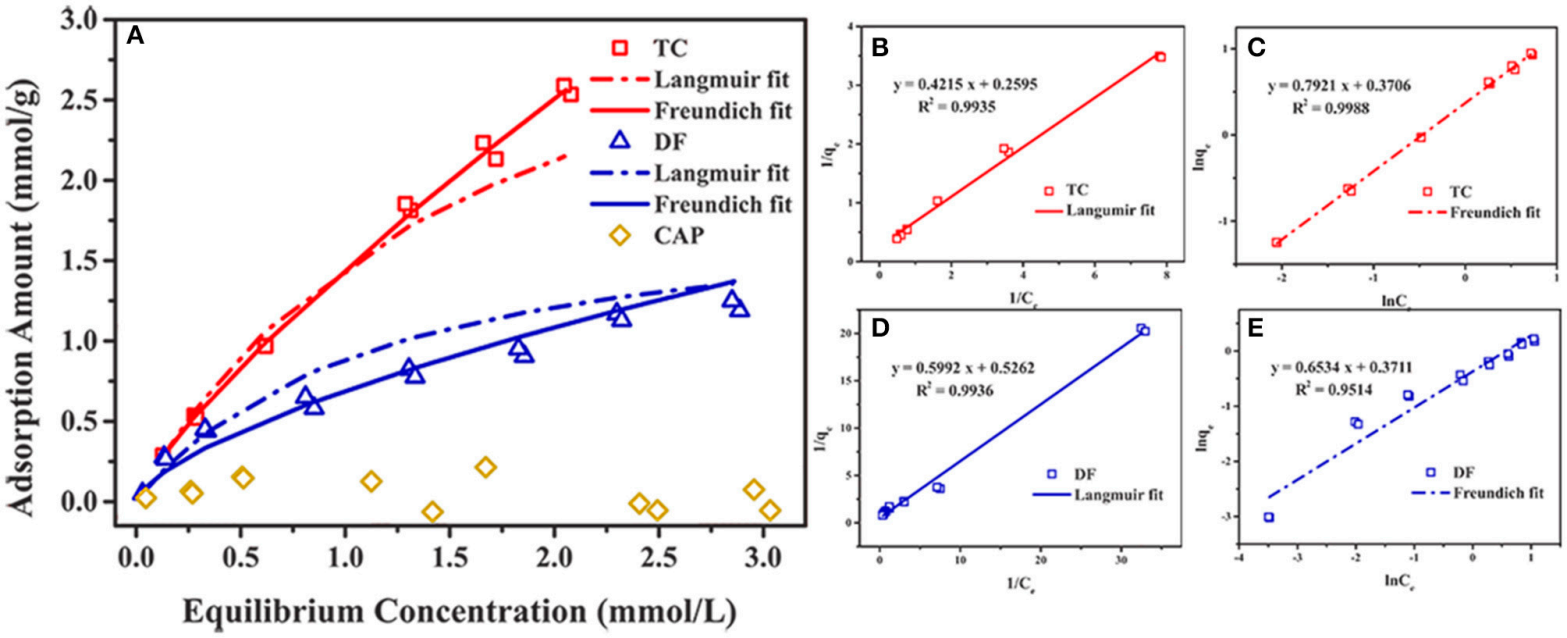
Adsorption of DF, TC, and CAP by Cl-LDH at room temperature **(A)**, the Langmuir and Freundlich fitting of the uptake of TC and DF on Cl-LDH **(B–E)**.

The adsorption isotherms of Cl-LDH on different initial concentrations of TC, DF, and CAP were shown in Figure 4A. With increasing concentrations, the adsorption amount of TC increased rapidly. When the initial concentration was 2.4 mmol/L (the equilibrium concentration of 1.3 mmol/L), the adsorption amount reached 1.82 mmol/g. And when the equilibrium concentration was 1.85 mmol/L (the initial concentration of 2.4 mmol/L), the maximum adsorption amount of DF reached saturation about 0.95 mmol/g. The adsorption amount of CAP on Cl-LDH had no obvious change with the increase of initial concentration, and when the equilibrium concentration was 2.39 mmol/L (the initial concentration 2.4 mmol/L), the maximum adsorption amount was only 0.02 mmol/g.

The uptake of TC and DF on Cl-LDH were fitted using both Langmuir and Freundlich sorption models. The Langmuir model assumed that the uptake of specific homogeneous surface by monolayer sorption without interaction between adsorbed ions, which could be written by the following equation (Huang et al., 2016):

$$\frac{1}{q_{e}}=\frac{1}{q_{max}k_{l}C_{e}}+\frac{1}{q_{max}}$$

in which *q*_*e*_ (mmol/g) and *q*_*max*_ (mmol/g) were the amount of adsorbed TC and DF at equilibrium time and the maximum theoretical adsorption capacity, respectively. *C*_*e*_ (mmol/L) stood for the equilibrium solution concentration, and *K*_*l*_ (L/mmol) referred to the adsorption equilibrium parameter. The parameters *q*_*max*_ and *K*_*l*_ could be obtained from the intercept and the slope of a plot of *1/q*_*e*_ against *C*_*e*_.

The Freundlich equation was developed empirically, with no theoretical basis. It was useful for describing the sorption of ions by chemical adsorption and surface precipitation reactions. This model, which was appropriate for heterogeneous systems, could be expressed by the following equation:

qe=KFCe1n

where *K*_*F*_ and 1/*n* were characteristic parameters related to the sorption capacity and sorption intensity of the system, respectively. The values of *K*_*F*_ and 1/*n* were calculated from a linear plot of ln*q*_e_ against ln*C*_e_.

Figures 4B–E illustrated the Langmuir and Freundlich fitting of the uptake of TC and DF on Cl-LDH, and the adsorption isotherm parameters for TC and DF were summarized in Table 1. It was obvious that the Langmuir fitting had a bigger *R*^2^-value than Freundlich fitting, indicating the DF adsorption onto the surface of the Cl-LDH was monolayer, in contrast to TC adsorption onto the surface of the Cl-LDH was multilayer. The fitted TC and DF maximum sorption capacities were 1.85 and 0.95 mmol/g, respectively. Gao et al. (2012) reported the maximum adsorption capacity that the removal of TC by graphene oxide is 0.65 mmol/g, and Li et al. (2010) reported the maximum adsorption capacity for TC by smectite is 0.40 mmol/g. Meanwhile, the complete different behavior between TC and DF uptake may reflected totally different mechanisms of their uptake on Cl-LDH the same experimental condition.

**Table 1:** Langmuir and Freundlich parameters for the TC and DF onto Cl-LDH.

**Adsorbent**	**Langmuir equation**			**Freundlich equation**		
	***q_*m*_* (mmol/g)**	***K_*L*_* (L/mmol)**	***R*^2^**	***K_*F*_* (mol^1-1/n^L^1/n^ g^−1^)**	***n***	***R*^2^**
TC	1.85	1.19	0.993	1.05	1.38	0.999
DF	0.95	1.14	0.996	1.53	0.69	0.983

### Cl-LDH sorption kinetics

**Figure 5:**
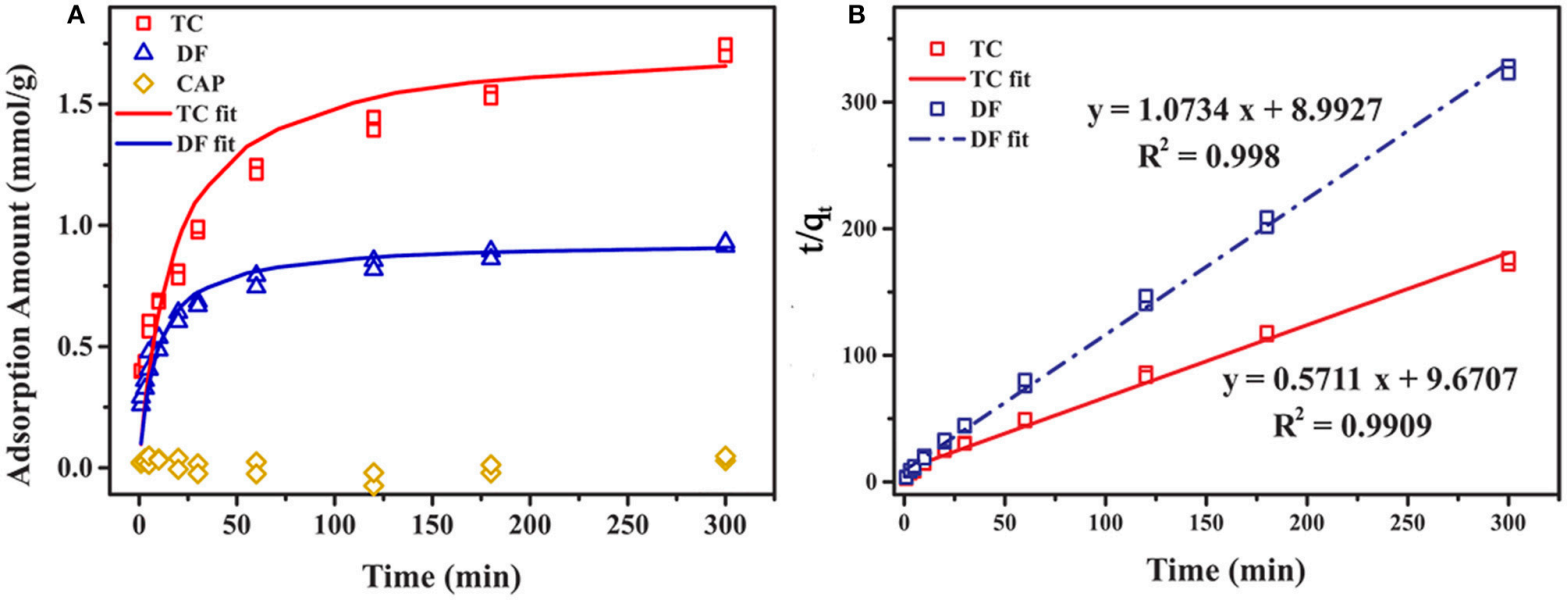
Adsorption amount of DF, TC, and CAP onto Cl-LDH as a function of time **(A)**, and the linear plot of pseudo-second-order model fit of DF and TC adsorption on Cl-LDH **(B)**.

The effect of contact time on the adsorption amount of Cl-LDH at room temperature was investigated, and the adsorption capacity of DF, TC, and CAP onto Cl-LDH as a function of time was depicted in Figure 5. With the increasing reaction time, the adsorption amount of TC and DF increased gradually then reaches saturation, while adsorption amount of CAP was small and showed no obvious change. The rate of TC and DF uptake was rapid in the first 60 min, where adsorption amount were 1.24 and 0.74 mmol/g for TC and DF, respectively. After that, the adsorption saturation was reached in 120 min, with the maximum adsorption amount of 1.72 and 0.91 mmol/g for TC and DF, respectively. At the initial stage, the high adsorption rate might be attributed to abundant vacant active sites on Cl-LDH surfaces available for adsorption. With a gradual reducing quantity of active sites, the electrostatic repulsive force played the leading role in the TC and DF loading on Cl-LDH. Thus, the adsorption of TC and DF slowed down, and finally the adsorption equilibrium was established.

In order to understand the mechanism of adsorption and evaluate the TC and DF adsorption performance of Cl-LDH adsorbent, the pseudo-first-order kinetic, and pseudo-second-order kinetic models were applied to analyze the dynamics adsorption experimental data. The kinetic models were expressed as follows (Ho and Mckay, 1998; Fu et al., 2015):

The pseudo-first-order kinetic model:

$$\log(q_{e}-q_{t})=\log q_{e}-\frac{k_{1}}{2.303}t$$

The pseudo-second-order kinetic model:

$$\frac{t}{q_{t}}=\frac{t}{q_{e}}+\frac{1}{k_{2}q_{e}^{2}}$$

where *k*_1_ (1·min^−1^) and *k*_2_ (g·mol^−1^·min^−1^) were the pseudo-first-order equilibrium rate constant and the pseudo-second-order equilibrium rate constant, respectively; *q*_1_ and *q*_2_ (mmol·g^−1^) were the TC and DF adsorption amount at equilibrium; *q*_*t*_ (mmol·g^−1^) was the amount of the TC and DF adsorbed at time *t*.

The experimental data could be well fitted with the pseudo-second-order model. The values of *q*_*e*_ and *k*_2_ could be calculated from the interception and slope of the linear equation of *t/q*_*t*_ against *t* (Figure 5). The parameters of pseudo-second-order kinetic for TC and DF were summarized in Table 2.

**Table 2:** Pseudo-second-order model constants for the TC and DF adsorption on Cl-LDH.

**Adsorbent**	**TC**			**DF**		
	***K*_2_ (g·mmol^−1^·min^−1^)**	***q_*e*_* (mmol/g)**	***R*^2^**	***K_2_* (g·mmol^−1^·min^−1^)**	***q_*e*_* (mmol/g)**	***R*^2^**
Cl-LDH	0.03	1.75	0.991	0.13	0.93	0.998

The adsorption capacities of TC and DF in the kinetic study were 1.75 and 0.93 mmol/g, respectively. The result was in good accordance with the results obtained from the experiment data (1.72 and 0.91 mmol/g for TC and DF). The effect of the contact time on the desorption of extent of TC and DF was also discussed in Supplementary Information.

### XRD analyses

**Figure 6:**
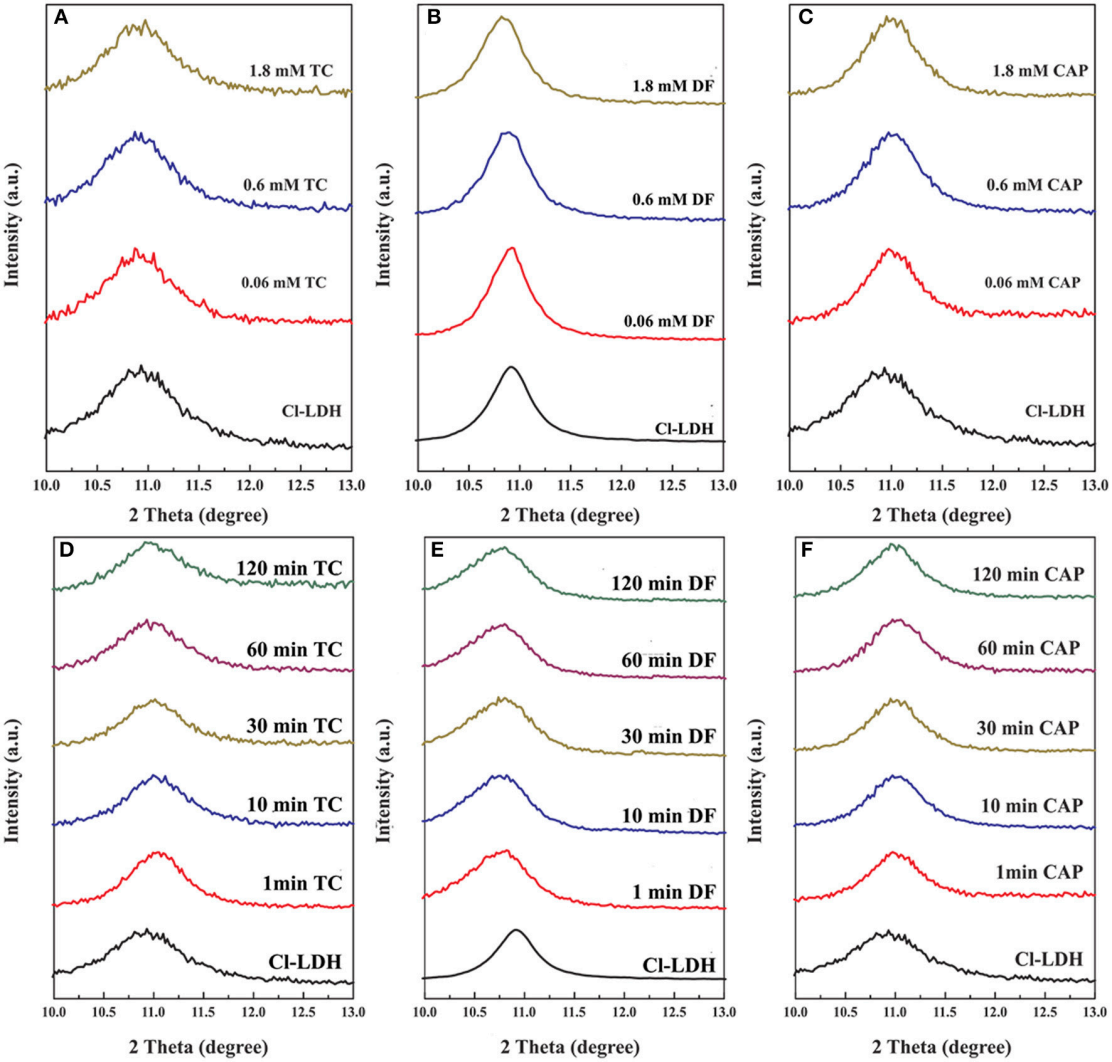
X-ray diffraction pattern of Cl-LDH before and after adsorption of TC, DF, and CAP. **(A–C)** Patterns of Cl-LDH after TC, DF, and CAP adsorption with different initial concentrations, respectively. **(D–F)** XRD patterns of Cl-LDH after TC, DF, and CAP adsorption at different reaction time, respectively.

The XRD patterns of Cl-LDH samples before and after adsorption of TC, DF, and CAP were shown in Figure 6. Spacing values of (003) remained approximately the same, indicating that anion exchange, especially exchanged with interlayer anion did not account for the high loading efficiency of TC. So the sorption of TC on Cl-LDH were all on the external surfaces. The *d*_003_ peak of Cl-LDH shifted toward smaller angle after the adsorption of DF, of different initial concentrations and different reaction time, as revealed by Figures 6B,E, suggesting the adsorption of DF was based on intercalation of Cl-LDH. Besides, no newly-appeared or no shift in peak was observed in CAP experiment, (Figures 6C,F) which meant the adsorption of CAP was mainly on the external surfaces of Cl-LDH or Cl-LDH tended not to adsorb CAP. The results of molecular simulation (Figure S2) was in good agreement with the XRD results.

### Zeta potential determination

**Figure 7:**
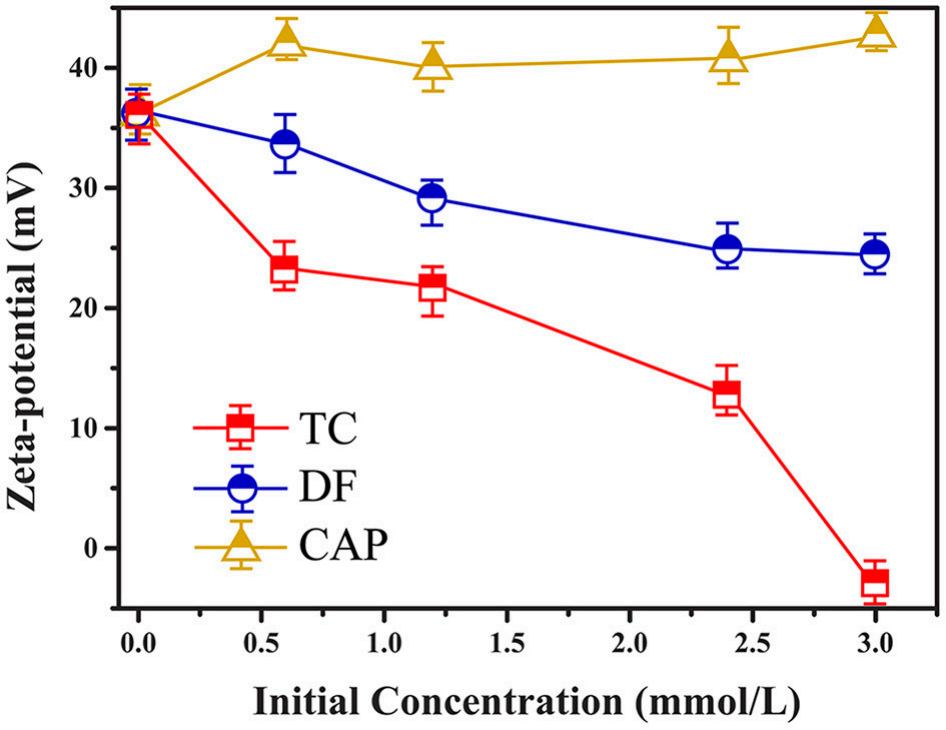
Zeta potential of Cl-LDH after TC, DF, and CAP adsorption.

Zeta potential was an indicator for the surface charge as well as the surface hydrophobicity, and the zeta potential of Cl-LDH before and after adsorption was illustrated by Figure 7. The Cl-LDH had a zeta potential of 36.2 mV (pH = 7), indicating positively charged surfaces. As the initial concentrations went up, there was a significant decrease of zeta potential during the adsorption of TC onto Cl-LDH, which was caused by the electrostatic attraction between TC and Cl-LDH. It agreed well with the results from XRD analysis of TC/Cl-LDH. The adsorption of TC onto Cl-LDH was a process of charge neutralization on the external faces. The zeta potential of DF/Cl-LDH showed a smaller decrease compared with TC/LDH. Combined with the result from XRD analysis, the adsorption of DF might probably be consisted of two parts: electrostatic attraction on the surface and intercalation into Cl-LDH layers. Nevertheless, the zeta potential of CAP/Cl-LDH remained almost the same after adsorption despite the increase of the initial concentrations, which reproved that the adsorption of CAP onto Cl-LDH tended to be poor.

### FTIR analyses

**Figure 8:**
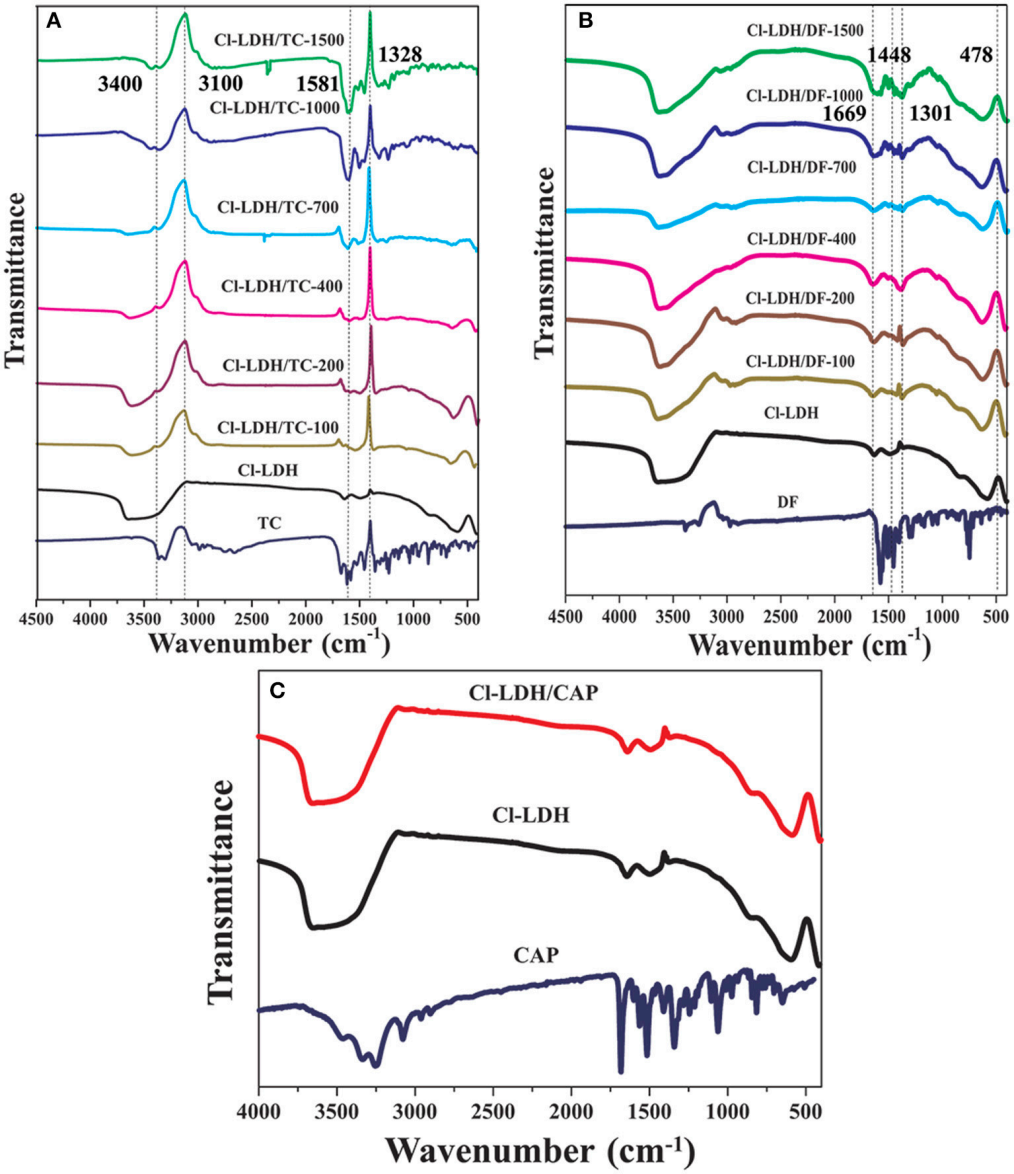
FTIR spectra of CL-LDH after adsorption of TC **(A)**, DF **(B)**, and CAP **(C)**.

The FTIR spectra of Cl-LDH samples before and after adsorption of TC, DF, and CAP were shown in Figure 8. In the spectra of Cl-LDH adsorbed TC, the broad peak located in the region of 3,100–3,400 cm^−1^ related to O–H stretching vibrations. The fingerprint peaks of TC range from 1,600 to 750 cm^−1^ emerge on the FTIR spectra of Cl-LDH after adsorption, which indicated that the TC had been effectively adsorbed on Cl-LDH. The band at 1,581.45 cm^−1^, which could be attributed (C = C) vibration in aromatic rings. The band at 1,328.21 cm^−1^ was due to the C–O stretching vibration of phenol group, as shown in Figure 8A. The DF phases primarily appeared in four characteristic bands: the peak at 1,669 cm^−1^ was due to the C = O stretching vibration, the peak of 1,448 cm^−1^ referred to the flexural vibration of the CH_2_, the peak of 1,301 cm^−1^ was attributed to the C–N extension, and the peak of 478 cm^−1^ corresponded to the C–Cl stretching vibration, as shown in Figure 8B. For CAP-adsorbed Cl-LDH, there showed no obvious characteristic vibration peak of CAP. It suggested CAP could not be adsorbed by Cl-LDH, as shown in Figure 8C.

## Conclusions

An Cl-LDH was synthesized, characterized, and examined for removal of the three chosen PPCPs (TC, DF, and CAP) from aqueous solution. According to XRD pattern, the prepared adsorbent had a typical LDH nanostructure. TC sorption on Cl-LDH followed the Freundlich type isotherm, and DF sorption on Cl-LDH followed the Langmuir type isotherm. The TC and DF sorption by Cl-LDH fitted the pseudo-second-order model well. The novel adsorbent shown a high adsorption capacity of TC and DF (1.82 and 0.95 mmol/g). The adsorption mechanism of PPCPs into Cl-LDH were proved to be different. The TC adsorption on Cl-LDH was accompanied by the electrostatic interactions between the negative charge of TC and the positive charge of Cl-LDH. The uptake of DF was attributed to negative exchange and electrostatic interaction. Cl-LDH did not adsorb CAP due to no electrostatic interaction. The molecular dynamic simulation further confirmed different configurations of three selected PPCPs were ultimately responsible for the uptake of PPCPs on Cl-LDH. The overall results of the present study suggested the efficient use of Cl-LDH for the removal of TC and DF from the aqueous solution.

## Author contributions

GL and LL designed the experiments. EL and CY carried out the experiments and analyzed the data. YL carried out the experiments. All authors discussed the results, EL, GL, and ZL wrote the paper. The manuscript has been reviewed and approved by all authors.

### Conflict of interest statement

The authors declare that the research was conducted in the absence of any commercial or financial relationships that could be construed as a potential conflict of interest. The reviewer WW, and handling Editor declared their shared affiliation.
