# Supplementary material for: The Interactions Between Three Typical PPCPs and LDH
Source: Front Chem. 2018 Mar 5;6:16. doi: 10.3389/fchem.2018.00016 (PMC5844944; doi:10.3389/fchem.2018.00016)
Supplement: Supplementary file 1 [file Presentation1.pdf]

Supplementary Information

The interactions between three typical PPCPs and LDH

Erwei Li[a], Guocheng Lv[a,*], Libing Liao[a,*], Zhaohui Li[b], Chengxue Yang[a]

[a]Beijing Key Laboratory of Materials Utilization of Nonmetallic Minerals and Solid Wastes, National Laboratory of Mineral Materials, School of Materials Science and Technology, China University of Geosciences, Beijing, China.

[b]Geosciences Department, University of Wisconsin–Parkside, Kenosha, WI, U.S.A.

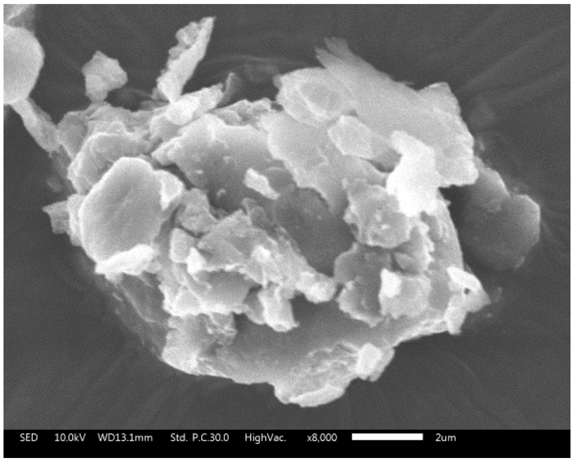

Fig S1. SEM images of as-prepared Cl-LDH samples

* Corresponding author: Email: guochenglv@cugb.edu.cn (G.L.); lbliao@cugb.edu.cn (L.L.)

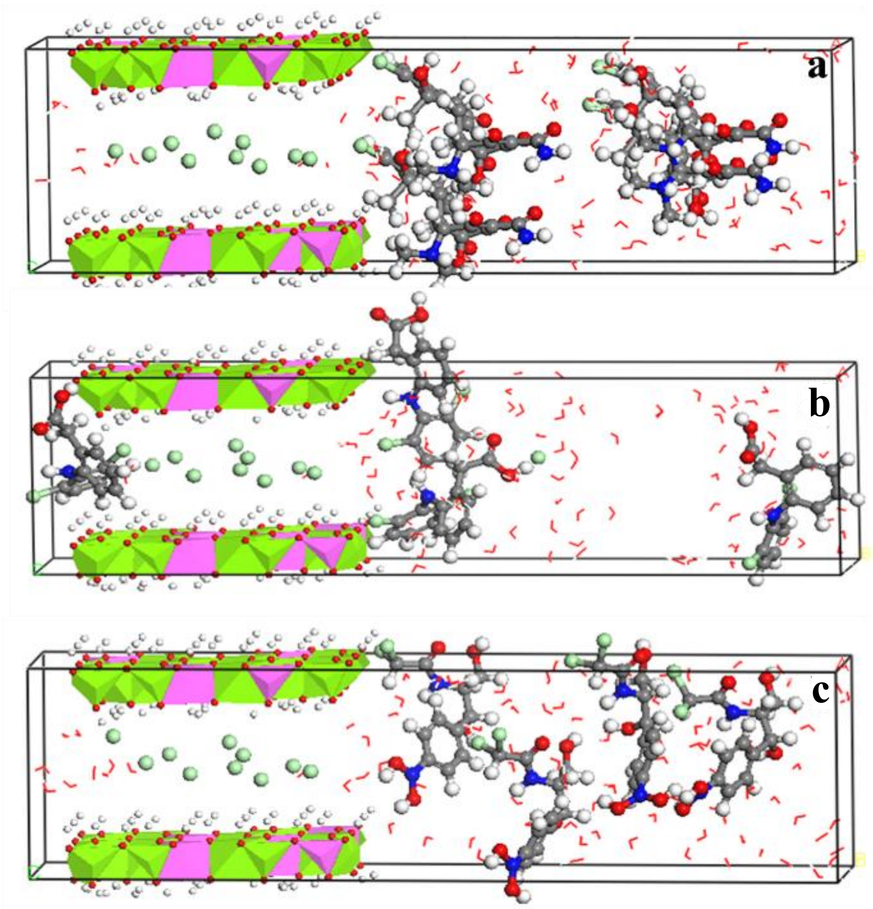

Fig. S2 Molecular dynamic simulation of adsorption of TC (a), CAP (b), and DF (c) onto Cl-LDH.

Molecular simulation was performed under the module ‘Forcite’ of Materials Studio 6.0 software to study the configuration of PPCPs in the surface of Cl-LDH. The unit cell (R-3) parameters were set at a=b=3.046 Å, c=22.78 Å, a=90, γ =90°, and b=120°. A series of 2×2×1 supercells were built. The established model was optimized geometrically. The temperature was set 298 K and the time was 1 ns with a time step of 1 fs. CLAYFF force field was used during simulation. After the system reached equilibrium, the NVT kinetic simulation was performed under the same time constant and temperature conditions. The data were collected on the last 300 ps for later analyses.

The simulation results for an initial concentration of 1.0 mmol/L intercalation showed how different adsorption methods of Cl-LDH removed three PPCPs. In the presence of water molecules, the equilibrium configuration of TC organic molecules and Cl-LDH was shown in Fig. S2a. It can be seen that the TC organic molecules were gathered together and clustered around the layer of Cl-LDH, which indicated that TC molecule did not enter the interlayer of Cl-LDH during adsorption. The higher adsorption amount of TC might be ascribed to electrostatic attraction. In the presence of water molecules, the equilibrium configuration of DF organic molecules and Cl-LDH was illustrated in Fig. S2b. Many kinds of arrangement ways of DF molecules could be seen in the configuration. The simulation result demonstrated that some DF molecules entered into the interlayer of Cl-LDH, and some of them were attached to the Cl-LDH surface. In aqueous solution, anion mineral Cl-LDH and no charged CAP molecule exhibited no electrostatic interaction, as shown in Fig. S2c.

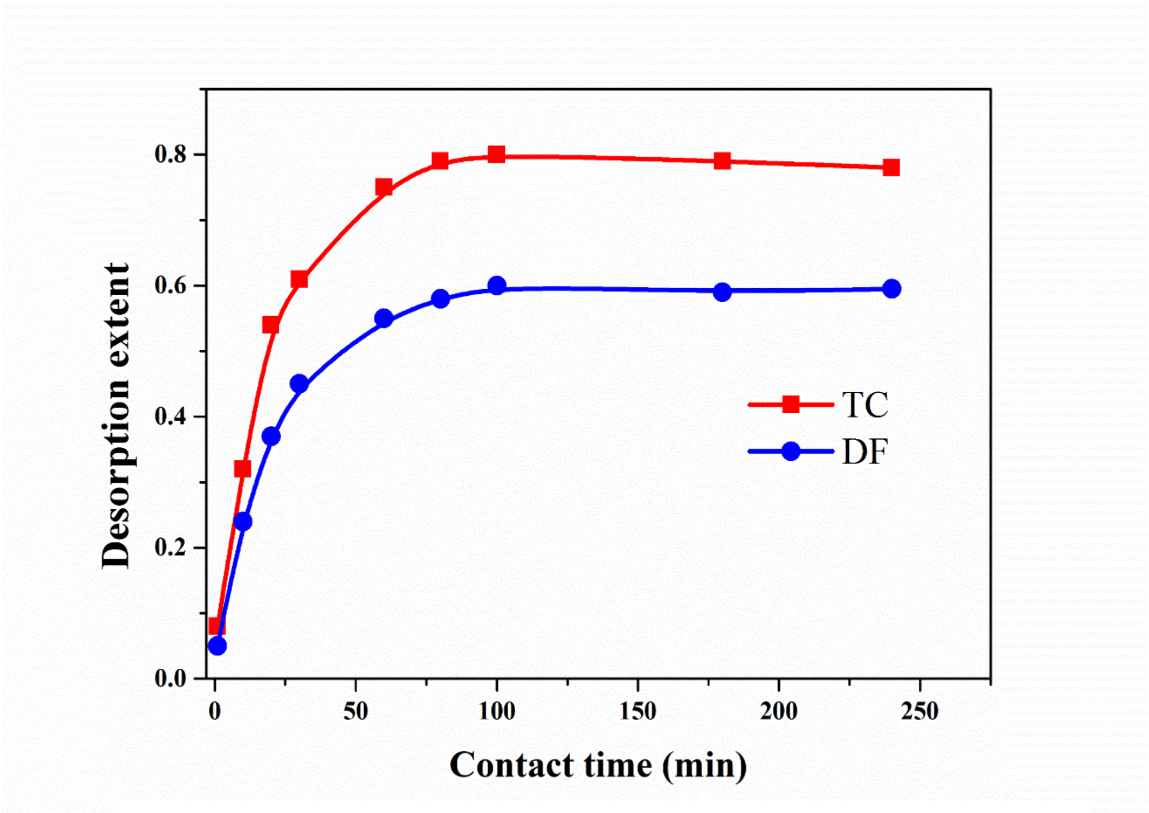

Fig. S3 Effect of the contact time on the desorption extent of TC and DF

0.015g Cl-LDH with saturated adsorption amount of PPCPs and 25mL 0.1 mol/L NaOH were added into a 50 mL centrifuge tube. The mixtures were shaken in a water bath with a speed of 150 rpm at room temperature for different time. After that, the mixtures were centrifuged by high speed centrifuge at 4500 rpm for 5 min. Then, the supernatant solutions were analyzed using the UV/VIS spectrophotometer to determine concentrations of PPCPs. Desorption rate was calculated based on the following equation:

$$\eta = \frac{c_1}{c_0} \times 100\%$$

η -- desorption rate of PPCPs;

c_0 -- initial concentration of PPCPs solutions in kinetic study, which was 2.4 mmol/L for both DF and TC;

c_1 -- final concentration of PPCPs after desorption

The results are as follws: the desorption extent of TC is about 80.5%, and that of DF is about 60.3%.
